# Factors affecting the intention of Iranian rural women to use medicinal herbs

**DOI:** 10.1186/s12906-023-03964-3

**Published:** 2023-05-27

**Authors:** Heshmatollah Saadi, Somayeh Jafari, Saeid Karimi

**Affiliations:** Department of Agricultural Extension and Education, Bu-Ali Sina University, Hamedan, Iran

**Keywords:** Medicinal herbs, Theory of Planned Behavior, Dissatisfaction with Modern Medicine, Rural Women, Iran

## Abstract

**Background:**

This study aimed to identify factors affecting the behavioral intention of Iranian rural women to use medicinal herbs. The research model was developed by integrating “dissatisfaction with modern medicine” into the “theory of planned behavior”.

**Methods:**

Data were collected through questionnaire from a sample of 260 Iranian rural women, which were randomly selected. The validity and reliability of the scale were confirmed using expert opinions and Cronbach’s alpha method, respectively.

**Results:**

Based on the results of structural equation modeling, attitude (β = 0.44; *p* < 0.01), subjective norms (β = 0.27; *p* < 0.01) and dissatisfaction with modern medicine (β = 0.11; *p* < 0.05) had significant positive effects on rural women’s intention to use medicinal herbs. In addition, subjective norms indirectly affected rural women’s intention to use medicinal herbs through attitude (β = 0.23; *p* < 0.01).

**Conclusions:**

Subjective norms was a key factor in determining the intention of Iranian rural women to use medicinal herbs, followed by attitude and dissatisfaction with modern medicine. Therefore, this study could contribute to our understanding on how the intention of Iranian rural women to use medicinal herbs was influenced by different factors.

**Supplementary Information:**

The online version contains supplementary material available at 10.1186/s12906-023-03964-3.

## Introduction

Medicinal herbs play a key role in ensuring the health of society due to their therapeutic and preventive properties. Medicinal herbs include to a wide range of plants that are used in the treatment and prevention of various diseases. Culture, laws and regulations, and scientific developments determine the diversity of plants used in different countries. medicinal plants are plants that are used entirely or partly, in fresh, dried, or processed forms, to diagnose, treat, prevent, or maintain the health of people, animals, or other plants [1]. International health entities have paid considerable attention to medicinal plants in recent years. The use of medicinal plants is growing rapidly worldwide [2, 3]. Today, 65–80% of the world’s population use medicinal plant products [1]. In developing countries, about 70% of people prefer to use medicinal plant products as their main source of health care [4, 5]. Given the diversity of plant species, the public culture, and the long history of Iranian traditional medicine, Iranians routinely use medicinal plants to treat and prevent various diseases [6]. According to studies, about 8000 plant species grow in Iran, many of which are considered medicinal species for various reasons. The prevalence of consumption of herbal medicines in different regions of Iran has been reported to be about 19–90% [6, 7]. Evidence suggests that Iranian women are more likely to use medicinal plants and traditional medicine than men [8, 9] and especially Iranian rural women have a relatively high tendency to use medicinal herbs [7].

Numerous theories and models have been developed to assess health-related intentions and behaviors. In this regard, the Theory of Planned Behavior (TPB) is among the most important and valid theories [10]. This theory provides a clear framework for examining the determinants of behavioral intention. Moreover, various tests performed at different spatial and temporal conditions have confirmed high theoretical strength, reliability, and validity of the TPB in predicting consumer behavior in various scientific areas including the use of medicinal plants [11, 12]. Based on the TPB, intention is the most important factor in determining whether or not to perform a particular behavior. In addition, people with more deliberate intentions are more likely to perform a particular behavior. In fact, behavioral intention shows the amount of an individual’s efforts to perform a behavior. This intention is determined by three main constructs including attitude, subjective norms, and perceived behavioral control. Attitude is the degree to which an individual has a desirable or undesirable assessment of a particular behavior. Subjective norms refer to perceived external pressures to display or avoid a particular behavior. These norms reflect people’s willingness/unwillingness to follow others. Perceived behavioral control reflects an individual’s perception of the difficulty of a particular behavior, and the degree to which he/she has control over the behavior. In other words, it refers to an individual’s belief in the adequacy of his/her abilities to perform a particular behavior. People with sufficient opportunities and resources who are confident about their abilities have stronger perceived behavioral control over their tasks. Attitude and subjective norms affect behavior through intention. In addition, perceived behavioral control influences behavior both directly and through intention [10, 13]. According to the TPB, people’s intention to use medicinal plants will not grow stronger unless they believe in valuable properties of these plants. In addition, influential people must encourage them to use medicinal plants, and authorities must provide people with necessary opportunities and resources to process these useful plants.

The TPB has widely been used in predicting health-related behaviors [14], supplementation consumption behaviors [15], and complementary and alternative drugs’ purchase intention [16, 17]; however, the researchers could not find sufficient evidence on factors affecting the use of medicinal plants by rural women in the literature. Therefore, the study used the TPB to identify factors affecting the behavioral intention of rural women to use medicinal plants. The findings are expected to help health care policymakers develop better educational programs and devise detailed plans to promote the use of medicinal plants.

Ajzen [10, 18] suggests that the TPB can be extended and modified by including additional constructs to capture more variance in behavioral intentions. Likewise, in this study, the construct of dissatisfaction with modern medicine is added to the TPB. Dissatisfaction is defined as the “negative affect about outcome unfavourability.” In other words, it signifies the failure to meet an individual’s expectations or needs. In this light, according to the expectation-confirmation theory [19], an individual forms either a positive or a negative affective valuation of an object; depending on such a valuation, he/she subsequently exhibits support behaviors or coping behaviors. Moreover, according to the theory of reasoned action, a positive affect leads to a continuation intention while dissatisfaction is followed by an intention towards discontinuation [10]. In the context of modern medicine, dissatisfaction refers to the degree to which people unfavorably perceive their modern medicine experiences. For instance, modern medicine fatigue and dissatisfaction reflect negative feelings and emotions that users experience when using modern medicine, feelings which could generate push effects on the users’ discontinuous use of modern medicine and make them switch to alternative medicines. Indeed, dissatisfaction with modern medicine has attracted the attention of many researchers, as it seems to influence the intentions of users to switch from modern medicine to alternative medicine. Based on previous studies, dissatisfaction with modern medicine (due to its high costs, the fear of severe side effects, and poor access to concomitantly suitable health care facilities) reinforces people’s tendency to resort to alternative medicine and is one of the main reasons behind the increased use of herbal medicines [20–24]. Especially, the evidence indicates that Iranian rural people tend to use medicinal plants due to their dissatisfaction with modern medicine, which is caused by the low effectiveness of chemical drugs, their side effects and lack of trust in them [25]. In other words, the more dissatisfied people are with modern medicine, its services, and its effects, the greater will likely be their intention to use medicinal plants.

**Fig. 1:**
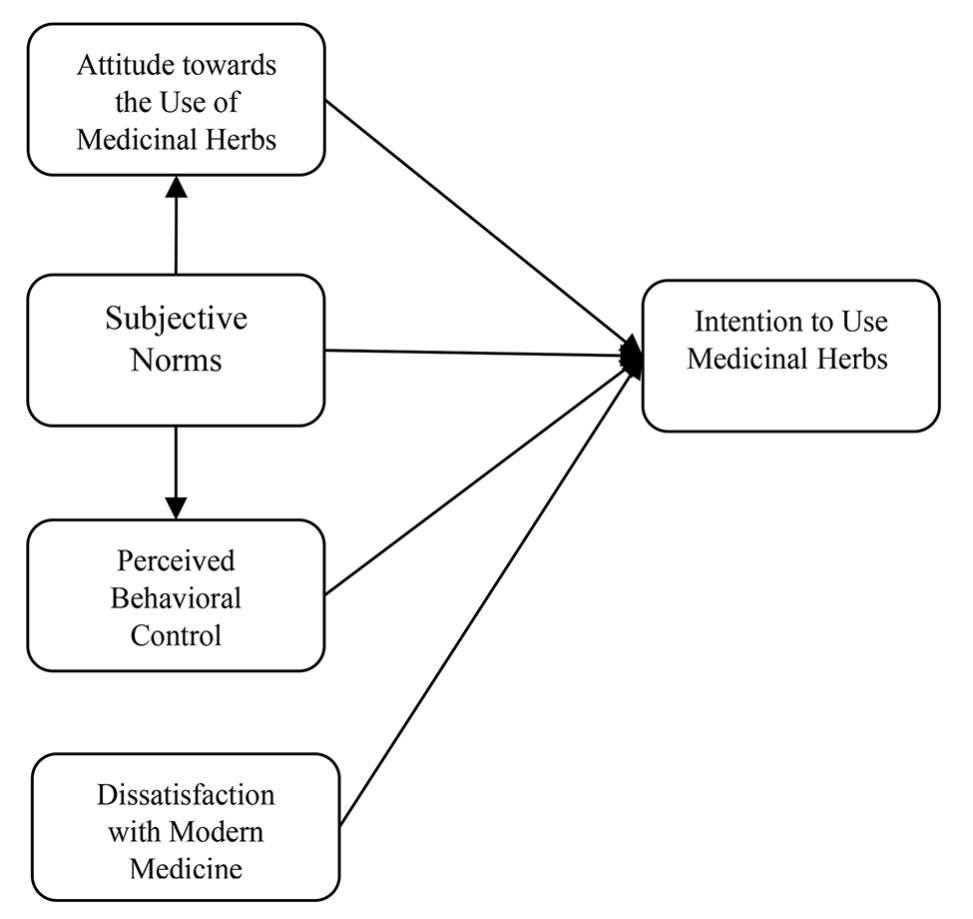
Theoretical research model

Based on the above discussion, the present study was conducted to examine factors affecting the intentions of Iranian rural women to use medicinal herbs based on the theory of planned behavior (Fig. 1).

## Research method

This cross-sectional descriptive study was carried out from late September to late October 2019. The study population consisted of all rural women living in Asadabad County (N = 3425) located in western Iran, regardless of their health status. The eligibility criteria for rural women to be selected in the present study included, (a) living at the selected villages, (b) being over the age of 18, (c), using medicinal herbs before, and (d) volunteering their participation in the study. A total of 260 individuals were randomly selected as the sample using Bartlett’s table. The data were collected using a structured questionnaire, which was designed based on previous studies. The questionnaire was completed by the second author through a face-to-face interview. Participants were verbally informed of the nature and the purpose of the research, but the study did not require approval by an ethics committee or a recognized institutional review board because it was a simple observational evaluation exclusively for academic research purposes with full anonymity of participants. The questionnaire consisted of two parts. The first part included the participants’ demographic information (e.g. age, educational qualifications, marital status, and income level). The second part consisted of the theoretical constructs of TPB and the construct of dissatisfaction with modern medicine.

### Measures

The measures used in this study were adopted from previously validated studies [7, 26]. The content validity of the scale was assessed by a panel of experts in terms of the necessary, relevancy, and clarity of items. The expert panel consisted of five specialists in the fields of medicinal herbs, health education, rural development, extension education, and psychology. Minor modifications and changes were made according to the panel’s recommendations. Subsequently, face validity was pilot tested by 25 rural women who were not included in the main sample. They were asked to comment on the wording and readability of the questionnaire. The pilot test indicated that there was no further need to modify the content of the survey. All constructs demonstrated good reliability with values of Cronbach’s alpha above 0.7 (α ≥ 0.70). The questionnaire items are listed in Additional file 1.

All five constructs were measured based on a five-point Likert scale (ranging from 1 = *strongly disagree* to 5 = *strongly agree*). To measure attitude a seven-item scale was used. Sample item includes: “In my opinion, the use of medicinal herbs is beneficial”. To measure subjective norms a four-item scale was used. Sample item includes: “Most people who are important to me recommend the use of medicinal herbs and traditional medicine”. Perceived behavioral control was measured by a four-item scale. Sample item includes: “I have easy access to medicinal herbs”.

To measure intention to use medicinal plants a three-item scale was used. Sample item includes: “I will consume medicinal herbs, if I experience health problems in the future”. Finally, dissatisfaction with modern medicine was measured using six items. Sample item includes: “Modern medicine cannot fulfill my expectation of treatment”.

### Data analysis

To test the research hypotheses, the partial least squares structural equation modeling (PLS-SEM) was used in SmartPLS 3.2.8. This software is one of the most popular structural equation modeling software worldwide. This method includes two steps. In the first step, the measurement model is assessed to test the validity and reliability of the research variables. In the second step, the structural model is assessed by testing the hypotheses, calculating variance explained by the endogenous variables, and determining the predictive power of different variables. This statistical method is not sensitive to the normality of the data and can well predict and support complex models with several direct and indirect relationships [27].

## Results

The mean age of the participants was 35.15 years. The majority of them were married (84%) and housewives (55%), with a mean family size of four people. The largest part of the women had high school diplomas (23.1%) and the lowest number of them were illiterate (3.1%). In this study, medicinal herbs were defined as any leaves, roots, flowers or any part of a plant or plant extract that was being used to preserve or recover health without medical advice or supervision. The majority of the participants (71%) used low to relatively high amounts of medicinal plants over the past year. The most commonly used medicinal herbs included Sattar, Spearmint, Green tea, Basil, Cinammon, Ginger, Borage, Flixweld, Cumin and Garlic. The findings indicated that the majority of participants used the medicinal plants in the form of infusions (53%) and distillates (20%) and only 3% of them used medicinal plants in the form of raw herbs. Most of the respondents (84%) obtained medicinal plants from the area surrounding their villages or traditional herbal shops.

Table 1 shows the mean and standard deviation of the variables, and correlation coefficients among them. There are significant positive correlations among the main research variables.

**Table 1:** Means, standard deviations, and correlations for all variables

Variables	Mean	SD	1	2	3	4	5	6	7
1. Age	35.15	11.03	-						
2. Education	4.55	1.55	− 0.34^**^	-					
3. Family size	4.00	1.28	0.25^**^	− 0.16^*^	-				
4. Dissatisfaction with modern medicine	3.17	0.67	0.05	− 0.17^*^	0.03	-			
5- Attitude	3.61	0.63	0.12	− 0.01	0.01	0.52^**^	-		
6- Subjective norms	3.55	0.78	0.01	− 0.05	0.02	0.39^**^	0.59^**^	-	
7- Percived behavioral control	3.53	0.73	0.05	− 0.20	0.12	0.42^**^	0.59^**^	0.39^**^	-
8. Intention	3.62	0.79	0.07	− 0.07	0.11	0.36^**^	0.71^**^	0.57^**^	0.40^**^

*Note*: *p < 0.05; **p < 0.01

Education was measured by a number from 1 to 7 (1— Illiterate 4—University degree)

### Measurement model

In this section, the reliability and validity of the measurement model were first assessed. Composite reliability (CR) and Cronbach’s alpha (CA) were used to assess the reliability of the research constructs. To assess the convergent and discriminant validity of the scale, Average Variance Extracted (AVE) and the Heterotrait-Monotrait Ratio of Correlations (HTMT) were used, respectively [28]. As shown in Table 2, all Cronbach’s alpha and CR values are greater than 0.7. In addition, AVE values for all research constructs are greater than 0.5; therefore, the reliability and convergence validity of the measurement model are desirable.

**Table 2:** Cronbach’s alpha coefficient, CR, AVE, R^2^ and Q^2^ for the latent research variables

Construct	Cronbach’s alpha	CR	AVE	R^2^	Q^2^
**1- Behavioral intention**	0.79	0.88	0.70	0.54	0.35
**2- Attitude**	0.84	0.85	0.51	0.27	0.13
**3- Subjective norms**	0.81	0.87	0.63	-	-
**4- Percived behavioral control**	0.74	0.84	0.56	0.15	0.08
**5- Dissatisfaction with modern medicine**	0.73	0.83	0.42	-	-

According to Table 3, all HTMT values are smaller than 0.90; thus, the discriminant validity of the measurement model is also confirmed.

**Table 3:** Discriminant validity based on HTMT criterion

Construct	1	2	3	4
**1- Behavioral intention**	-			
**2- Attitude**	0.84			
**3- Subjective norms**	0.70	0.63		
**4- Behavioral control**	0.56	0.67	0.50	
**5- Dissatisfaction with modern medicine**	0.61	0.70	0.53	0.49

### Structural model

After testing the measurement model and confirming its validity and reliability, the structural research model was assessed. This model helps researchers test the research hypotheses. T-statistic, effect size (*f*^*2*^), coefficient of determination (R^2^), and path coefficient (β) were calculated to assess the structural model. According to Cohen, *f*^*2*^ values of 0.02, 0.15, and 0.35 represent small, medium, and large effect sizes, respectively. A coefficient of determination (R^2^) determines the effect of an exogenous variable on an endogenous variable. R^2^ values of 0.19, 0.33, and 0.67 indicate weak, moderate, and strong determination, respectively. As presented in Table 3, the obtained R^2^ values indicate that the model can explain 54%, 27%, and 15% of the variance of variables of intention, attitude, and perceived behavioral control, respectively. In addition, Stone-Geisser’s Q² values are positive for latent variables; hence, the predictive power of the structural model is acceptable. A goodness of fit (GOF) indicator was used to assess the goodness of fit of the model. GOF values of 0.1, 0.25, and 0.36 imply weak, moderate, and strong model fit, respectively [29]. This indicator is calculated using the following formula:

$$GOF = \sqrt {\overline {{R^2}} *\overline {Communality} }$$

The calculated GOF value (0.42) indicates the strong fit of the overall research model.

**Fig. 2:**
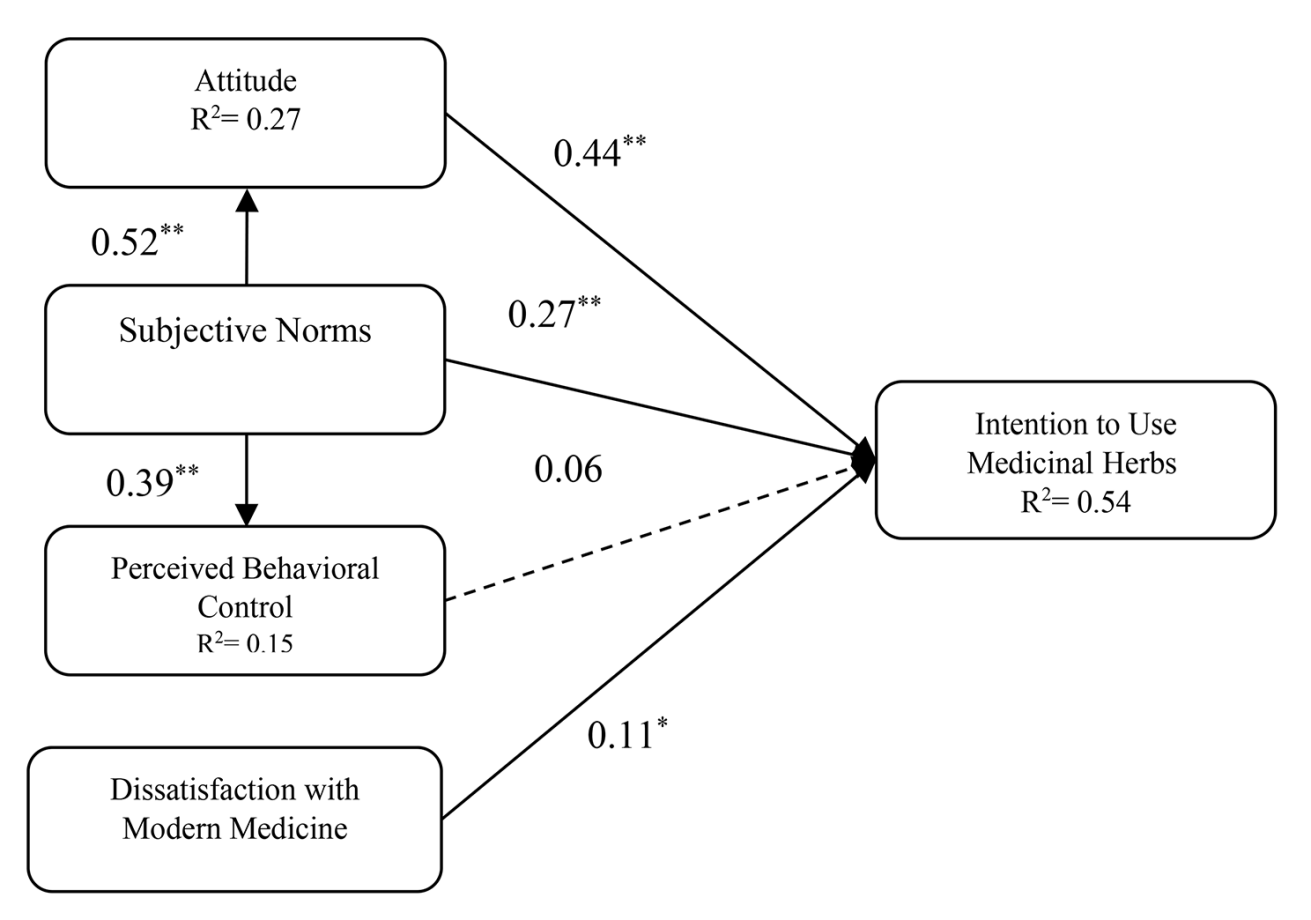
Results of structural model *Note*: Dashed lines indicate non-significant relationships; solid lines indicate significant relationships; **p<0.01, *p<0.05

As presented in Table 4; Fig. 2, the bootstrapping analysis results confirmed the significance of the relationships of attitude and behavioral intention *(β* = 0.44; *p* < 0.01), and subjective norms and behavioral intention (*β* = 0.27; *p* < 0.01); therefore, these relationships were confirmed. The effect sizes of attitude→ intention (*f*^*2*^ = 0.21) and subjective norms→intention (*f*^*2*^ = 0.11) were medium and small, respectively. No significant relationship was found between perceived behavioral control and behavioral intention (β = 0.06; *p* > 0.05); thus, this relationship was not supported. Subjective norms were found to have significant relationships with variables of attitude (*β* = 0.52; *p* < 0.01) and perceived behavioral control (β = 0.39; *p* < 0.01); therefore, these relationships were confirmed. In addition, dissatisfaction with modern medicine had a significant positive relationship with behavioral intention (*β* = 0.11; *p* < 0.05). Besides their direct relationship with behavioral intention, subjective norms also were indirectly associated with behavioral intention through attitude (β = 0.23; *p* < 0.01). Finally, perceived behavioral control was not significantly related to behavioral intention; hence, the mediating role of this variable was not examined (Table 4; Fig. 2).

**Table 4:** Direct, indirect, and total effects of the structural equation model

Path			Path coefficient (β)	*f* ^*2*^	Result		
**Direct effect**							
Attitude	→	Intention	0.44^**^	0.21		Supported	
Subjective norms	→	Intention	0.27^**^	0.11		Supported	
Behavioral control	→	Intention	0.06	0.00		Not supported	
Subjective norms	→	Attitude	0.52^**^	0.38		Supported	
Subjective norms	→	Behavioral control	0.39^**^	0.18		Supported	
Dissatisfaction with modern medicine	→	Intention	0.11^*^	0.02		Supported	
**Indirect effect**							
Subjective norms → Attitude	→	Intention	0.23^**^			Supported	
**Total effect**							
Subjective norms	→	Intention	0.53**				

(*P* < 0.01**, *P* < 0.05*)

## Discussion

This study aimed to identify factors affecting the intention of Iranian rural women to use medicinal herbs, using the framework of the TPB. Rural women’s attitude towards the use of medicinal herbs was found to have the greatest direct positive effect on their behavioral intention. In other words, a positive evaluation of the benefits of medicinal herbs by rural women reinforces their intention to use these herbs. Attitude reflects an individual’s way of thinking, values, beliefs, and feelings, as well as his/her reactions to the surrounding environment; thus, this factor can directly influence one’s behavioral intention. This is especially important in rural areas given the common values and beliefs about health-related properties of medicinal plants and the religious and spiritual philosophy behind the use of these plants [30]. Also, this finding is consistent with the results of research by other researchers [12, 26, 30]. In addition, this is consistent with the meta-analysis study by McEachan et al. [14] which demonstrated that attitude towards a behavior is the strongest predictor of intention in various health behaviors.

In addition, subjective norms had significant positive effects on rural women’s intention, attitude, and perceived behavioral control regarding the use of medicinal herbs. In addition, it had the greatest total effect on behavioral intention (βtotal = 0.53). In other words, positive attitude of influential people (e.g. family members, friends, and doctors) towards the use of medicinal herbs will subconsciously influence rural women’s attitude towards these herbs. This will in turn improve rural women’s behavioral control, and will reinforce their intention to use medicinal plants. Some researchers argue that social norms/influence affect people’s attitudes and behavioral intentions to use traditional medicine and medicinal herbs [17, 30]. This is of great importance in rural areas due to the socio-cultural condition of these regions, high levels of trust and cohesion (i.e. high levels of social capital), especially among women, and profound significance of social norms [31]. Accordingly, Sereshti and Azari observed that most women use herbal substances on the advice of their friends and families [32]. This is indicative of the important role played by social pressure as well as views of reference groups in the attitude and behavior of Iranian rural women.

Based on the results, perceived behavior control had no significant effect on the participants’ intention to use medicinal herbs. In other words, perceived difficulty in using medicinal herbs and having necessary opportunities and resources did not significantly affect rural women’s intention to use these herbs. This finding is inconsistent with the results of previous studies [12, 23]. This difference is probably due to several reasons. For example, medicinal herbs are mostly gathered and processed by rural women almost without any expenditure. In addition, the participants stated that they can fully control the use of medicinal plants. Accordingly, Fishbein and Ajzen state that researchers do not need to assess perceived behavioral control, when the behavior is voluntarily controlled [18]. Further research is required to better understand this phenomenon.

Dissatisfaction with modern medicine directly influenced rural women’s behavioral intention. When people perceive that modern medicine is not as effective as expected and does not meet their medical needs satisfactorily, they probably switch to alternative medicines, such as traditional medicine and medicinal herbs, to meet their needs and expectations [33]. Many researchers consider dissatisfaction with modern medical services as a major determinant of tendency towards the use of medicinal herbs [20–24].

## Study limitations

This study has interesting findings; however, some limitations have emerged. Given the cross-sectional nature of the present study, the structural equation modeling results do not prove the casualty of the aforementioned relationships. In this regard, researchers can design longitudinal studies to assess cause-and-effect relationships. Considering the limitations of self-report questionnaires, researchers can use other methods (e.g. observation and field surveys) along with questionnaires to measure variables in future studies. Moreover, we did not consider the possible effects of respondent characteristics such as health and socioeconomic status. Because these factors may affect individuals’ tendency to use medicinal herbs, they should be controlled for in future research. In addition, this study examined rural women’s intention instead of their actual behavior. Although behavioral intention is the closest predictor of actual behavior, it does not always lead to actual behavior. Therefore, scholars are suggested to design longitudinal studies in order to investigate rural women’s actual behavior in using medicinal herbs. Finally, the extended TPB was found to account for 54% of the variance in intention to use medicinal herbs, implying that other factors such as knowledge and satisfaction may also influence rural women’s intentions. These factors can be included in the TPB to improve its explanatory power.

## Conclusion

This study contributes to the literature on the use of medicinal plants through its application of an extended TPB. It hereby offers theoretical implications to produce a better understanding of the mechanism underlying Iranian rural women’s intention to use medicinal herbs. The results can provide insight for future scholars exploring in-depth views on the intention to use medicinal herbs among rural women in Iran.

Hence, it is suggested that healthcare institutions and practitioners educate local people, especially rural women, on the possible benefits of medicinal plants and encourage their use to promote a culture of using such treatments. As more people have begun to use medicinal plants and their products, the attitudes toward them have started to change. As the results indicated, positive beliefs and attitudes are significant influencers of people’s intentions, and this will encourage the use of medicinal plants and their products in the future. In addition, healthcare policymakers and practitioners need to establish access to accurate and reliable information about medicinal herbs for rural women to ensure that they can engage in safe and effective self-care. Given the significant effect of attitudes and social norms on rural women’s intention to use medicinal plants, accurate information can be provided to these women and their families, relatives, and friends through mass media concerning the importance and benefits of these plants as well as their proper consumption, effectiveness, and potential side effects. This prevents the transmission of inaccurate data, and thereby modifies the pattern of consumption of these herbs among the general public, especially among rural women. In addition, holding continuous educational courses for medicinal herb sellers is another suggestion of the present study. Overall, this study identifies the factors that influence the intention of Iranian rural women to use medicinal herbs and, hopefully, provides important implications for health practitioners and policymakers when making plans to promote the proper use of medicinal herbs among Iranian rural women.

## Electronic supplementary material

Below is the link to the electronic supplementary material.

**Additional file 1:** Supplemental Table 1

## Data Availability

The data that support the findings of this study are available from Bu-Ali Sina University but restrictions apply to the availability of these data, which were used under license for the current study, and so are not publicly available. Data are however available from the authors upon reasonable request and with permission of the corresponding author.
