# Supplementary material for: Factors affecting the intention of Iranian rural women to use medicinal herbs
Source: BMC Complement Med Ther. 2023 May 27;23:170. doi: 10.1186/s12906-023-03964-3 (PMC10224271; doi:10.1186/s12906-023-03964-3)
Supplement: Supplementary file 1 — Additional file 1: Supplemental Table 1 [file 12906_2023_3964_MOESM1_ESM.docx]

Supplemental Table for

***Factors affecting the intention of Iranian Rural Women to Use Medicinal Herbs***

**Supplemental Table 1.** The questionnaire based on the theory of planned behavior.

| Construct | Item |
| --- | --- |
| **Intention** | I will use medicinal herbs, if I experience health problems in the future. |
|  | I would recommend relatives, friends and colleagues to use medicinal herbs. |
|  | I would encourage others to use medicinal herbs. |
| **Attitude** | I think the use of medicinal herbs is beneficial. |
|  | I think the use of medicinal herbs is wise. |
|  | I think medicinal herbs are safe. |
|  | I think medicinal herbs have very few side effects. |
|  | Medicinal herbs are more effective compared to chemical medications in healing diseases. |
|  | Medicinal herbs can offer patients benefits that chemical medicine cannot**.** |
|  | Overall, I prefer using medicinal herbs over chemical medications. |
|  | I think the use of medicinal herbs is beneficial. |
| **Subjective norms** | Most people who are important to me recommend the use of medicinal herbs and traditional medicine. |
|  | Most of the people who are important to me approve of the use of medicinal plants to heal diseases. |
|  | Most people who are important to me think I should use medicinal plants to heal diseases |
| **Perceived behavioral control** | I have easy access to medicinal herbs. |
|  | I can overcome my difficulty in using medicinal herbs. |
|  | The procedures to use medicinal herbs are easy. |
|  | The cost of using medicinal plants is lower compared to chemical medications. |
|  | I can share my knowledge and experience of medicinal plants with others. |
|  | I have the ability to decide whether to choose medicinal plants. |
| **Dissatisfaction with modern medicine** | I think that modern medicine cannot cure many diseases.  I do not feel better after receiving modern medicine. |
|  | Modern medicine cannot fulfill my expectation of treatment. |
|  | The side effects of modern medicines are more than their benefits. |
|  | I am very dissatisfied with my experiences of using modern medications. |
|  | I regret using modern medications. |
